# Mating, births, and transitions: a flexible two-sex matrix model for evolutionary demography

**DOI:** 10.1007/s10144-018-0615-8

**Published:** 2018-06-01

**Authors:** Esther Shyu, Hal Caswell

**Affiliations:** 1 Biology Department MS-34, Woods Hole Oceanographic Institution, Woods Hole, MA 02543 USA; 2 Institute for Biodiversity and Ecosystem Dynamics, University of Amsterdam, PO Box 94248, 1090 GE Amsterdam, The Netherlands

**Keywords:** Birth matrix-mating rule, BMMR, Demography, Matrix population models, Sex-biased harvest, Two-sex models

## Abstract

Models of sexually-reproducing populations that consider only a single sex cannot capture the effects of sex-specific demographic differences and mate availability. We present a new framework for two-sex demographic models that implements and extends the birth-matrix mating-rule approach of Pollak. The model is a continuous-time matrix model that explicitly includes the processes of mating (which is nonlinear but homogeneous), offspring production, and demographic transitions and survival. The resulting nonlinear model converges to exponential growth with an equilibrium population composition. The model can incorporate age- or stage-structured life histories and flexible mating functions. As an example, we apply the model to analyze the effects of mating strategies (polygamy or monogamy, and mated unions composed of males and females, of variable duration) on the response to sex-biased harvesting. The combination of demographic complexity with the interaction of the sexes can have major population dynamic effects and can change the outcome of evolution on sex-related characters.

**Electronic supplementary material:**

The online version of this article (10.1007/s10144-018-0615-8) contains supplementary material, which is available to authorized users.

## Introduction

Models of sexually-reproducing populations that consider a only single sex (typically females) implicitly assume that both sexes have identical vital rates and that the availability of the neglected sex (typically males) does not affect fertility (Pollard 1974; Caswell 2001; Iannelli et al. 2005). In reality, both these assumptions are frequently violated. Males and females often differ significantly in terms of fertilities and mortalities (Kuczynski 1932; Pollak 1990; Jenouvrier et al. 2010), developmental schedules (Caswell 2001), behavioral interactions (Rankin and Kokko 2007), dispersal patterns (Miller et al. 2011), and selective harvest pressures (Ginsberg and Milner-Gulland 1994). Additionally, ecological, environmental, and evolutionary changes may alter the most limiting sex over time (Hardy 2002; Miller and Inouye 2011). One-sex models miss, and cannot explore, important components of population dynamics in all these cases. Here, we introduce a flexible framework that can explore many of these factors.

In response to growing concerns over discrepancies in male and female reproductive rates (Karmel 1947), early dynamical models with sex structure were introduced in the late 1940s. Pollard (1948) used coupled Lotka integral equations, considering female births to males and male births to females in order to reconcile the growth rates of both sexes. Kendall (1949) introduced a system of ordinary differential equations for males and females (and later, married couples), and was the first to incorporate nonlinear interactions between the sexes via a mating term. Subsequent models considered other nonlinear mating functions (Pollard 1974; Yellin and Samuelson 1974) and couple dissolution through death and divorce (Hadeler et al. 1988).

Extensions to age-structured populations were made by Fredrickson (1971) and Hoppensteadt (1975), who allowed birth and death rates, as well as couple formation and divorce rates, to depend on age and sex. Hadeler later included the age (i.e., duration) of married pairs (Hadeler et al. 1988) and maturation delays (Hadeler 1993). Age-structured mating functions have similarly been proposed (Martcheva and Milner 2001). Many of these models use continuous-time equations that incorporate age structure through coupled McKendrick–von Foerster partial differential equations (Fredrickson 1971; Keyfitz 1972; Hoppensteadt 1975; Hadeler 1989, 1993), though discrete-time two sex matrix models have also been developed (Caswell and Weeks 1986).

A powerful conceptual approach to two-sex models was proposed by Pollak (1986, 1987, 1990) and called the birth matrix-mating rule (BMMR) model. It proposes that mating, births, and life cycle transition processes repeat periodically, one after the other. It contains three main components:

1. A mating rule function that gives the number of matings $u_{ij}$ between males of age *i* and females of age *j*.
2. A birth matrix whose entries $b_{ij}$ are the expected number of male and female offspring produced by a mating of a male of age *i* and a female of age *j*.
3. Sex-specific mortality schedules, which project surviving individuals to the next age class, or, in our generalization, include other stage-specific life cycle transitions.

BMMR is a useful approach for describing two-sex populations because it can specify age (and, more generally, stage) structure over all parts of the life cycle. This structure, in turn, can have significant effects on two-sex population dynamics (e.g., Sundelöf and Åberg 2006, where the addition of size-specific birth functions affects growth rate and reproductive output) and recommended management strategies (e.g., Ginsberg and Milner-Gulland 1994, where incorporating age-specific fecundity changes the outcomes of sex-biased harvest).

Conceptually appealing as it is, the BMMR model has yet to be fully incorporated into the framework of stage-classified matrix population models. Our goal here is to do so, providing a general model that can be adapted to a wide range of life cycles and mating strategies. We do so by a novel extension of periodic matrix models to continuous time, based on transition rate matrices. In this paper, we focus on ecological implications of the model, but the approach can also be applied to study the evolution of sex-related traits using methods from adaptive dynamics (Shyu and Caswell 2016a, b).

## Model development

For reasons that will become apparent, we have incorporated sex structure, stage structure, and life cycle processes into a continuous-time, rather than a discrete-time matrix population model. The mating, birth, and transition processes from the BMMR framework are described by separate rate matrices. The mating process introduces nonlinearity into the model because reproduction depends on the relative proportions of males and females, of appropriate stages, in the population. This dependence can be flexibly modeled by generalized weighted mean mating functions, which satisfy the biological criteria of sexual reproduction (Iannelli et al. 2005). The resulting BMMR matrix models are nonlinear but homogeneous (i.e., frequency-dependent). Models of this form generally converge to an exponential growth rate and a stable stage frequency distribution (Martcheva 1999).

### Incorporating sex and stage structure

The model classifies individuals into stages based on age, developmental state, sex, reproductive status, or other variables of interest. Stage densities are projected forward in time by a projection matrix that contains the demographic rates characterizing survival, reproduction, and transitions between stages. The properties of this projection matrix provide information about the population as a whole, thereby linking individual-level life cycle information (i.e., the stage-specific vital rates in the matrix entries) to population-level properties important for ecology and evolution (e.g., growth rates or stage distributions).

A population with *s* stages is described by a $s \times 1$ population vector $\mathbf{n}(t)$, the entries of which are the densities of each stage at time *t*. In a two-sex population, $\mathbf{n}(t)$ would contain male stages, female stages, and mated stages (unions) that could include married couples or breeding harems.

For example, the population vector for a two-sex population with mating adults and nonmating juveniles could have the form:

1 $$\begin{aligned} \mathbf n(t) = \begin{pmatrix} \text {juvenile males}\\ \text {adult males} \\ \text {juvenile females}\\ \text {adult females}\\ \text {adult unions} \end{pmatrix} \end{aligned}$$

The dynamics of the population vector are given by a system of ordinary differential equations

2 $$\begin{aligned} \frac{{\text {d}}\mathbf {n}}{{\text {d}}t} = \mathbf {A}[\mathbf {n}] \; \mathbf {n}(t) \end{aligned}$$

where the entries of $\mathbf {A}$ are either transition rates or rates of offspring production, and we have indicated that they may depend on the population vector.

### Incorporating the BMMR processes

The BMMR framework incorporates mating, birth, and transition processes. We describe each of these processes by a separate matrix:

1. The mating (union formation) process, where adult males and females organize into reproductive unions, is described by the matrix $\mathbf {U}$.
2. The birth process, where unions produce new offspring, is described by the matrix $\mathbf {B}$.
3. The transition process, which includes other life cycle events like mortality, maturation, or divorce, is described by the matrix $\mathbf {T}$.

Other life cycle processes can be included with additional matrices.

A discrete-time model would incorporate these processes as a periodic matrix product (Caswell and Shyu 2012). For example, the product $\mathbf{T} \mathbf{B} \mathbf{U}$ would describe mating, followed by the production of offspring from the matings, followed by survival and transitions of the resulting individuals. In continuous-time, we conceptualize the processes as occurring simultaneously. It can be shown (Appendix 1) that the projection matrix $\mathbf {A}$ in the continuous-time matrix model (Eq. 2) is the average of the transition rate matrices, e.g.,:

3 $$\begin{aligned} \frac{{\text {d}}\mathbf {n}}{{\text {d}}t}&= \frac{1}{3} \left( \mathbf {T} + \mathbf {B} + \mathbf {U} \right) \mathbf {n}(t) \nonumber \\&=\mathbf {A} \mathbf {n}(t) \end{aligned}$$

### Modeling the mating process

The mating process, as described by the union formation matrix $\mathbf {U}$, depends on the relative numbers of males and females in the population, not all of which may be mature enough or available for breeding (Pollard 1974; Iannelli et al. 2005). As a result, $\mathbf {U}$ depends on the population’s sex and stage composition, making $\mathbf {A}$ a function of the population vector $\mathbf {n}(t)$.

The total mating function $M(\mathbf {n})$ gives the rate of union formation (total number of unions formed per unit time in a population of composition $\mathbf {n}$). This embodies the mating rule in the BMMR framework. Here, “unions” refers to any mated, reproducing units in the population, including both one-to-one male-female pairs and harems with multiple individuals of the one sex.

We convert the total mating function into the per capita mating rates $U_m(\mathbf {n})$ and $U_f(\mathbf {n})$ (the average mating rates per available males *m* or females *f* respectively),

4 $$\begin{aligned} U_m(\mathbf {n})&= \frac{M(\mathbf {n})}{m}\end{aligned}$$

5 $$\begin{aligned} U_f(\mathbf {n})&= \frac{M(\mathbf {n})}{f} . \end{aligned}$$

The total population mating rate is $M = U_m m = U_f f$.

**Fig. 1:**
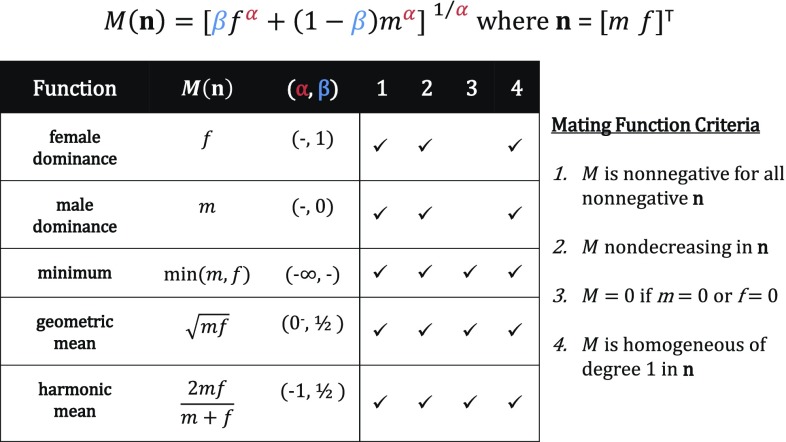
Mating functions from the generalized weighted mean family (Eq. 6) with a check to indicate which of the biologically desirable mating function criteria they satisfy

Many commonly used mating functions are generalized weighted means (Hölder means) of the form

6 $$\begin{aligned} M(\mathbf {n}) = \left[ \beta f^\alpha + (1-\beta ) m^\alpha \right] ^{1/\alpha} \end{aligned}$$

where $\beta$ and $\alpha$ are constants; $0\le \beta \le 1$, $\alpha < 0$ (Hadeler 1989; Bessa-Gomes et al. 2010; Iannelli et al. 2005). Figure 1 shows several generalized weighted mean mating functions and biologically desirable criteria that they satisfy (McFarland 1972; Pollard 1974; Yellin and Samuelson 1974).

If multiple male and female stages interbreed to form different types of unions, stage-specific mating preferences can also be integrated into this mating function (Shyu and Caswell 2016b; see  Appendix 4).

It is difficult to distinguish between mating functions in populations where the sex ratio does not vary significantly (Keyfitz 1972). However, recent empirical studies (Miller and Inouye 2011) support the harmonic mean as a mating function. Because the harmonic mean satisfies reasonable biological criteria for mating functions (Caswell and Weeks 1986; Iannelli et al. 2005), and captures the qualitative properties of other generalized means, we will use a harmonic mean mating function for our analyses.

### Frequency-dependent dynamics

The mating process often depends on the relative frequencies, rather than absolute abundances, of males and females. As a result, although the mating function is nonlinear, it is homogeneous of degree 1 in $\mathbf {n}$. That is:

7 $$\begin{aligned} M(c\mathbf {n}) = c M(\mathbf {n}) \end{aligned}$$

for any positive constant *c*.

As a result, the per capita mating functions (Eqs. 4 and 5) are homogeneous of degree 0 in $\mathbf {n}$, so that:

8 $$\begin{aligned} U_m(c\mathbf {n})&= U_m(\mathbf {n}) \nonumber \\ U_f(c\mathbf {n})&= U_f(\mathbf {n}) \end{aligned}$$

If all entries in the projection matrix $\mathbf {A}$ in Eq. 3 are also homogeneous of degree 0, the system is said to be frequency-dependent. This means that $\mathbf {A}$ can be written as a function of the population frequency vector:

9 $$\begin{aligned} \mathbf {p} = \frac{\mathbf {n}}{ \Vert \mathbf {n} \Vert } \end{aligned}$$

where $\Vert \mathbf {n} \Vert$ is the 1-norm of $\mathbf {n}$.

Frequency-dependent models of this type usually converge asymptotically to an equilibrium population structure $\hat{\mathbf {p}}$ (the stable stage distribution) in which all stage frequencies are constant (e.g., see Yellin and Samuelson 1974; Hadeler 1989; Martcheva 1999). The population then grows or decays exponentially at a long-term growth rate given by the dominant eigenvalue $\lambda$ of $\mathbf {A}[\hat{\mathbf {p}}]$.

To find the equilibrium stage distribution $\hat{\mathbf {p}}$ and population growth rate $\lambda$, it is sufficient to consider the dynamics of $\mathbf {p}(t)$. It can be shown (Appendix 2) that:

10 $$\begin{aligned} \frac{d\mathbf {p}}{dt} = \left( \mathbf {I}_s - \mathbf {p} \mathbf {1}^{{\tiny \mathsf T}}\right) \mathbf {A}[\mathbf {p}] \; \mathbf {p} \end{aligned}$$

where $\mathbf {I}_s$ is a $s \times s$ identity matrix and $\mathbf {1}^{{\tiny \mathsf T}}$ is a $1 \times s$ vector of ones. One can integrate Eq. 10 with a numerical differential equation solver until population frequencies converge to $\hat{\mathbf {p}}$. Then $\lambda$ is the dominant eigenvalue of $\mathbf {A}[\hat{\mathbf {p}}]$. The dominant right eigenvector $\mathbf {w}$ of $\mathbf {A}[\hat{\mathbf {p}}]$ is proportional to the stable stage distribution $\hat{\mathbf {p}}$.

## A 5-stage BMMR matrix model

**Fig. 2:**
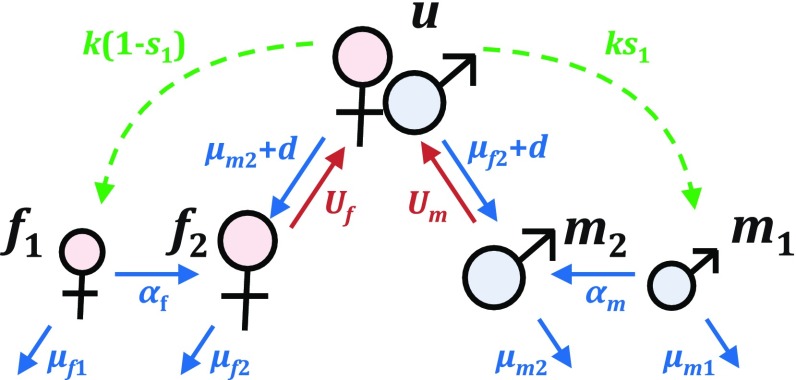
Life cycle diagram for a 5-stage population with juvenile males $m_1$ and juvenile females $f_1$, adult males $m_2$ and adult females $f_2$, and reproducing unions *u*. The functions and parameters shown here, as described in Table 1, appear in the union formation matrix $\mathbf {U}$ (Eq. 14) (red), birth matrix $\mathbf {B}$ (Eq. 15) (green), or transition matrix $\mathbf {T}$ (Eq. 16) (blue). From Shyu and Caswell (2016a)

We now present an example of a BMMR matrix model with five stages: juvenile males $m_1$ and juvenile females $f_1$, adult males $m_2$ and adult females $f_2$, and reproducing unions *u* that consist of one adult male and one adult female. Single adult males and females interact to form unions, which then produce new juvenile offspring (Fig. 2). A summary of the variables, parameters, and matrices in this model is provided in Table 1.

As in Eq. 1, we write the population vector as

11 $$\begin{aligned} \mathbf {n}(t) = \begin{pmatrix} m_1&m_2&f_1&f_2&u \end{pmatrix}^{{\tiny \mathsf T}}\end{aligned}$$

Using the harmonic mean mating function, the total and per capita mating functions are

12 $$\begin{aligned} M(\mathbf {n})= \frac{2 m_2 f_2}{m_2 + f_2} \nonumber \\ U_m(\mathbf {n})= \frac{2 f_2}{m_2 + f_2} \nonumber \\ U_f(\mathbf {n})= \frac{2 m_2}{m_2 + f_2} \end{aligned}$$

Again, we consider the life cycle in terms of mating, birth, and transition processes, which are described by matrices $\mathbf {U}$, $\mathbf {B}$, and $\mathbf {T}$ respectively.

1. The union formation matrix $\mathbf {U}$ contains the per capita mating functions from Eq. 12.
  13 $$\begin{aligned} \mathbf {U}(\mathbf {n})= & {} \begin{pmatrix} 0 &{} 0 &{} 0 &{} 0 &{} 0\\ 0 &{} -U_m(\mathbf {n}) &{} 0 &{} 0 &{} 0 \\ 0 &{} 0 &{} 0 &{} 0 &{} 0\\ 0 &{} 0 &{} 0 &{} -U_f(\mathbf {n}) &{} 0 \\ 0 &{} \frac{1}{2}U_m(\mathbf {n}) &{} 0 &{} \frac{1}{2}U_f(\mathbf {n}) &{} 0 \end{pmatrix} \end{aligned}$$
  14 $$\begin{aligned}\quad \quad = & {} \begin{pmatrix} 0 &{} 0 &{} 0 &{} 0 &{} 0\\ 0 &{} -\frac{M(\mathbf{n})}{m_2} &{} 0 &{} 0 &{} 0 \\ 0 &{} 0 &{} 0 &{} 0 &{} 0\\ 0 &{} 0 &{} 0 &{} -\frac{M(\mathbf{n})}{f_2} &{} 0 \\ 0 &{} \frac{M(\mathbf{n})}{2m_2} &{} 0 &{} \frac{M(\mathbf{n})}{2f_2} &{} 0 \end{pmatrix} \end{aligned}$$
  Note that $U_m$ and $U_f$ must halved in the last row of $\mathbf {U}$ to avoid double counting the unions formed from both male and female partners.
2. The birth matrix $\mathbf {B}$ contains *k*, the average reproductive rate of a union, and the primary sex ratio $s_1$, the proportion of offspring that are male.
  15 $$\begin{aligned} \mathbf {B} = \begin{pmatrix} 0 &{} 0 &{} 0 &{} 0 &{} k s_1 \\ 0 &{} 0 &{} 0 &{} 0 &{} 0 \\ 0 &{} 0 &{} 0 &{} 0 &{} k(1-s_1) \\ 0 &{} 0 &{} 0 &{} 0 &{} 0 \\ 0 &{} 0 &{} 0 &{} 0 &{} 0 \end{pmatrix} \end{aligned}$$
3. The transition matrix $\mathbf {T}$ contains the male mortality rates ($\mu _{m1}$ for juveniles, $\mu _{m2}$ for adults) and female mortality rates ($\mu _{f1}$ for juveniles, $\mu _{f2}$ for adults), the rates of maturation from juveniles to adults ($\alpha _m$ for males, $\alpha _f$ for females), and the divorce rate *d* (rate at which the male-female pair bond breaks). Note that unions may also dissolve due to partner death, and that union dissolution through both death and divorce returns surviving males and females to the single adult stages.
  16 $$\begin{aligned} \mathbf {T} = \begin{pmatrix} -(\mu _{m1}+\alpha _m) &{} 0 &{} 0 &{} 0 &{} 0 \\ \alpha _m &{} -\mu _{m2} &{} 0 &{} 0 &{} \mu _{f2} + d\\ 0 &{} 0 &{} -(\mu _{f1}+\alpha _f) &{} 0 &{} 0 \\ 0 &{} 0 &{} \alpha _f &{} -\mu _{f2} &{} \mu _{m2} + d\\ 0 &{} 0 &{} 0 &{} 0 &{} -(\mu _{m2} + \mu _{f2} + d) \end{pmatrix} \end{aligned}$$

The advantages of the continuous-time formulation are apparent at this point. The rates of maturation, mortality, and divorce in Eq. 16 combine in a simple additive fashion. Transition *probabilities* would not combine additively; they would involve sums of products of conditional probabilities, over all the possible events. The number of these products increases dramatically when more stages, and hence more mating combinations, are included. See Shyu and Caswell (2016b) and Appendix 4 for incorporation of multiple mating stages in the continuous-time model.

As per Eq. 3, the average of these three matrices is the continuous-time projection matrix $\mathbf {A}[\mathbf {n}]$. The corresponding equation for the proportional structure (Eq. 10) is thus:

17 $$\begin{aligned} \frac{d\mathbf {p}}{dt} = \left( \mathbf {I}_s - \mathbf {p} \mathbf {1}^{{\tiny \mathsf T}}\right) \frac{1}{3} \left( \mathbf {T + B + U[\mathbf {p}]} \right) \mathbf {p} \end{aligned}$$

where $\mathbf{U}$ is given by Eq. 14, $\mathbf{B}$ is given by Eq. 15, and $\mathbf{T}$ is given by Eq. 16.

**Fig. 3:**
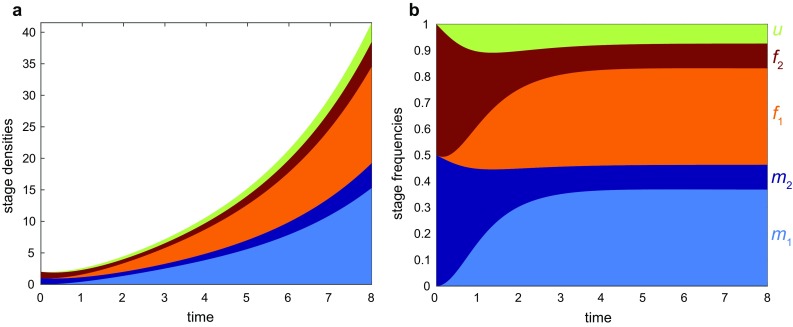
Dynamics of the 5-stage BMMR model with monogamous unions and no harvest. The population consists of juvenile males $m_1$ and juvenile females $f_1$, adult males $m_2$ and adult females $f_2$, and reproducing unions *u*. *a)* Growth of the population density vector $\mathbf {n}$ (11), where dynamics are given by (3). *b)* Convergence of the population frequency vector $\mathbf {p}$ (9), where dynamics are given by (17). Parameters are fixed at $\mu _{m1} = \mu _{f1} = 0.5, \mu _{m2}=\mu _{f2} = 0.1,\alpha _m=\alpha _f = 0.5, s_1= 0.5, k=20, d=0.1,h=1$, $E = 0$.

As shown in Fig. 3, the population vector $\mathbf {n}$ ultimately grows exponentially, while the frequency vector $\mathbf {p}$ ultimately converges to the constant distribution of stages $\hat{\mathbf {p}}$. To determine the equilibrium stage distribution $\hat{\mathbf {p}}$, we integrated Eq. 17 with the Matlab ODE45 differential equation solver until population frequencies converged (e.g., until vector entries do not change significantly over consecutive integration intervals).

The Matlab code used for all the following examples is provided in the Electronic Supplementary Material.

**Table 1:** A summary of the variables, parameters, matrices, and population properties in the 5-stage BMMR matrix model

*Matrices and vectors*	
$\,\mathbf {A}$	Projection matrix
$\,\mathbf {B}$	Birth matrix (15)
$\,\mathbf {T}$	Transition matrix (16)
$\,\mathbf {U}$	Union matrix (14)
$\,\mathbf {n}$	Population density vector (11)
$\,\mathbf {p}$	Population frequency vector (9)
$\,\hat{\mathbf {p}}$ or $\mathbf {w}$	Equilibrium stage structure
*Population properties*	
$\,\lambda$	Long-term population growth rate, dominant eigenvalue of $\,\mathbf {A}[\hat{\mathbf {p}}]$
$\,s_1$	Primary sex ratio (proportion of offspring that are born male)
$\,s_2$	Secondary sex ratio (proportion of adults that are male)
$\,f_1, f_2$	Juvenile, adult female density
$\,m_1, m_2$	Juvenile, adult male density
$\,u$	Union (mated pair) density
*Life cycle parameters*	
$\,\alpha _m, \alpha _f$	Male, female maturation rates
$\,d$	Divorce rate (rate at which a male-female pair bond breaks)
$\,h$	Average harem size
$\,k$	Union reproductive rate
$\,\mu _{f1}, \mu _{f2}$	Juvenile, adult female mortality rates
$\,\mu _{m1}, \mu _{m2}$	Juvenile, adult male mortality rates
$\,M$	Total mating rate (total unions formed per time)
$\,U_m, U_f$	Per capita mating rates (4) and (5)
*Harvest parameters*	
$\,E$	Total harvest rate in (18)
$\,s_h$	Harvested sex ratio (proportion of harvest that targets males) in Eq. 18

## Mating systems and the effects of sex-biased harvest

As an example of the use of the two-sex model, we consider the effectes of sex-biased harvesting. In sport or trophy hunting, harvest is often male-biased and significantly exceeds natural mortality (Festa-Bianchet 2003; Milner et al. 2007). Age or size bias is also common, as larger or older males with well-developed adult characteristics (e.g., large antlers or horns) are usually the most desirable targets. This unnatural selection may alter population structure, reproductive strategies, body morphology, and developmental timing (e.g., Ashley et al. 2003; Festa-Bianchet 2003; Allendorf and Hard 2009). The population response depends on multiple demographic factors, including the mating system.

The mating system determines how males and females organize for breeding and is thus a key component of two-sex population structure (Emlen and Oring 1977). Some species form monogamous, one-to-one pair bonds between males and females. Other species have polygynous mating systems in which one male mates with multiple females (e.g., lions, deer, seals), or, more rarely, polyandrous systems where one female mates with multiple males (e.g., jacanas, pipefish). Unions formed by mating may be transient and limited to a single breeding episode (e.g., lek systems) or may persist over multiple breeding seasons (e.g., lion harems) or even until partner death (e.g., albatrosses and other species with high mate fidelity) (Cézilly and Danchin 2008).

These factors motivate the use of a demographic two-sex model in analyzing harvest effects. To this end, we will use our BMMR matrix framework to explore the effect of mating systems on the response to sex-biased harvest. A range of mating systems will be approximated by varying two model parameters, *d* and *h* (Table 2).

- The divorce rate *d*. This is a measure of union transience. Unions with higher values of *d* are more likely to dissolve after a given mating, while unions with lower values of *d* are more likely to persist over multiple breeding interactions.
- The harem size *h*. This is a measure of polygamy. Unions with $h=1$ are monogamous and consist only of one-to-one male-female pair bonds, while unions with $h>1$ are polygamous groups of size $h+1$. As polyandrous mating systems are relatively rare (Cézilly and Danchin 2008), we will consider only the polygynous form of polygamy, where one male mates with *h* females.

**Table 2:** Mating systems corresponding to different values of the divorce rate *d* and harem size *h*

	Low *d* (persistent unions)	High *d* (transient unions)
$h = 1$ (monogamy)	Persistent pair bonds, high mate fidelity (e.g., albatross)	Serial pair bonds (e.g., humans, Emperor penguins)
$h> 1$ (polygyny)	Persistent harems (e.g., lion prides)	Leks, scramble competition (e.g., grouse, cod, horseshoe crabs)

Harvest strategies are characterized by the overall harvest rate *E* and the harvested sex ratio $s_h$ (proportion of the total harvest rate that targets males). Assuming that only adults are harvested, the adult mortality rates are modified by harvest as follows:

18 $$\begin{aligned} \mu _{m2}&\rightarrow \mu _{m2} + E s_h \nonumber \\ \mu _{f2}&\rightarrow \mu _{f2} + E (1 - s_h) \end{aligned}$$

To determine how various mating systems, as characterized by different values of *h* and *d*, respond to sex-biased harvest, we will examine harvest effects on the long-term population growth rate $\lambda$ and the secondary sex ratio $s_2$ (proportion of all adults that are male). We will assume that males and females have the same baseline vital rates, and that the primary sex ratio (proportion of males at birth) is 0.5. Thus, the main sex-specific differences we consider are sex-biased harvest pressures and male versus females roles within the polygynous mating systems.

### Monogamy ($h=1$)

Consider a monogamous two-sex population with juveniles and adults. The mating process forms unions that are one-to-one pair bonds of adult males and females. This scenario uses the rate matrices $\mathbf {U}$, $\mathbf {B}$, and $\mathbf {T}$ as given by Eqs. 14, 15, and 16 respectively, and the mating functions in Eq. 12. We vary the divorce rate *d* to explore the effects of transient vs. persistent pair bonds.

**Fig. 4:**
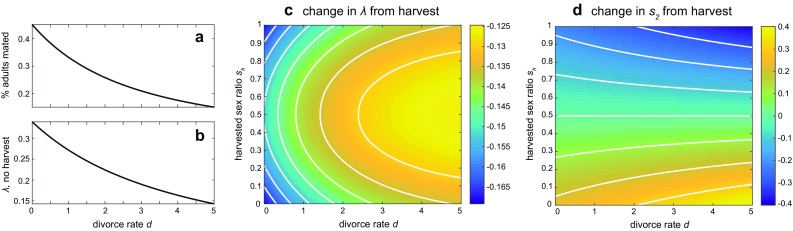
Population growth rates, structure, and responses to adult harvest in the monogamous ($h=1$) model. *a)* Proportion of mated adults (adults in stage *u*, rather than $m_2$ or $f_2$) for an unharvested population, and the *b)* corresponding population growth rates $\lambda$, as functions of the divorce rate *d*. *c)* The change in $\lambda$ and *d)* the change in secondary sex ratio $s_2$, when a proportion $s_h$ of harvest targets males. Without harvest, $s_2 = 0.5$ for all values of *d*. Other parameters are fixed at $\mu _{m1} = \mu _{f1} = 0.5, \mu _{m2}=\mu _{f2} = 0.1,\alpha _m=\alpha _f = 0.5, s_1= 0.5, k=20, E = 1$

As shown in Fig. 4a, the proportion of adults in the reproductive union stage (mated adults) decreases as *d* increases. The unharvested population growth rate $\lambda$ similarly decreases (Fig. 4b), because populations with more transient couples (fewer mated adults) cannot produce offspring as rapidly as populations with more persistent couples (more mated adults). Because males and females have the same baseline vital rates, the secondary sex ratio $s_2$ remains unbiased ($s_2=0.5$, not shown) for all values of *d*.

Figure 4c shows how increasingly sex-biased harvest strategies affect population growth. Both biased and unbiased harvest strategies most strongly reduce growth in populations with lower divorce rates, as adult mortality will also disrupt pair bonds. The greatest decreases in $\lambda$ occur when harvest is strongly sex-biased, i.e., $s_h$ is close to 0 (only females are harvested) or 1 (only males are harvested). This suggests that monogamous populations with high fidelity pair bonds will be the most impacted by sex-biased harvest, and that concentrating harvest on a single sex will more greatly reduce population growth.

Figure 4d shows how the same harvest strategies decrease the secondary sex ratio $s_2$ from equality. Unsurprisingly, male-biased strategies reduce $s_2$, female-biased strategies increase $s_2$, and unbiased strategies ($s_h=0.5$) have relatively minimal effect. Populations with greater divorce rates experience larger reductions in $s_2$, possibly due to their lower growth rates (Fig. 4a) being unable to replenish harvested individuals as rapidly.

### Polygyny ($h>1$)

Now consider a polygynous population that forms unions consisting of one male with a harem of females. Because the death or departure of a single female changes the harem’s size and reproductive rate, we must now account for multiple union (harem) stages.

The stage $u_i$ represents polygynous unions consisting of one male and a harem of *i* females. When *h* is the maximum harem size, the population vector contains *h* union stages, which range from a union with a harem of size 1 ($u_1$, equivalent to a monogamous couple) to a union with a harem of size *h* ($u_h$, the largest possible union):

19 $$\begin{aligned} \mathbf{n}(t) = \left( \begin{array}{cccccccc} m_1&m_2&f_1&f_2&u_1&u_2&\ldots&u_h \end{array} \right) ^{{\tiny \mathsf T}} \end{aligned}$$

Assume that when males and females mate, they form the largest possible union $u_h$. Their union formation rate is still given by the harmonic mean mating function Eq. 12, but with the number of single females now replaced by the number of prospective harems:

20 $$\begin{aligned} M(\mathbf {n})&= \frac{2 m_2 \frac{f_2}{h}}{m_2 + \frac{f_2}{h}} \nonumber \\ U_m(\mathbf {n})&= \frac{2 f_2}{h m_2 + f_2} \nonumber 
\\ U_f(\mathbf {n})&= \frac{2 m_2}{h m_2 + f_2} \end{aligned}$$

Note that the total union formation rate $M(\mathbf {n})$ is maximized when the sex ratio of single adults is

21 $$\begin{aligned} \frac{m_2}{m_2+f_2} = \frac{1}{1+\sqrt{h}} \end{aligned}$$

Thus, as the harem size *h* increases, a higher proportion of single females is needed to maximize the mating rate.

**Fig. 5:**
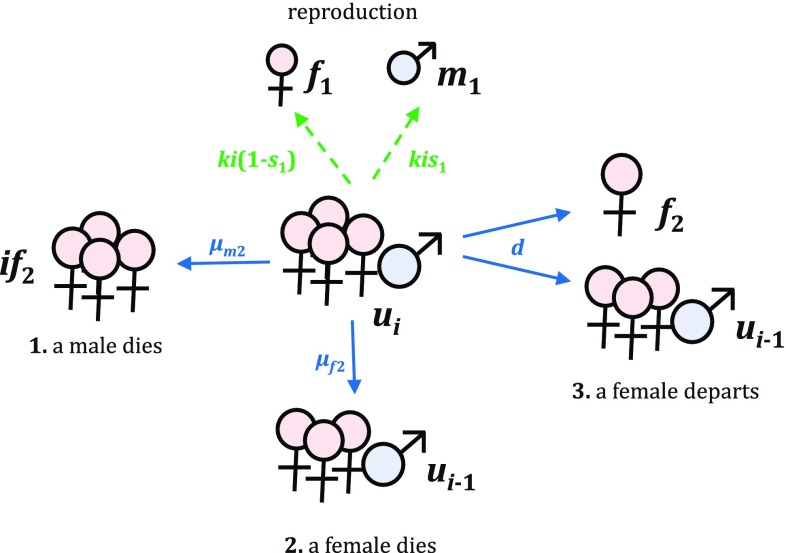
Reproduction and three possible transitions for $u_i$, a union with harem size *i*

If an individual female has a reproductive rate of *k*, a harem with *i* females has a total reproductive rate of *ik*; larger harems are thus more productive. Each union $u_i$, regardless of harem size, can change in three possible ways (Fig. 5):

1. The male harem leader dies (with mortality rate $\mu _{m2}$). This returns *i* adult females to the stage $f_2$.
2. A female harem member dies (with mortality rate $\mu _{f2}$). This shrinks the union from $u_i$ to $u_{i-1}$. For the union $u_1$, which has only one female, $u_{i-1} = u_0$ corresponds to the single adult male stage $m_2$ (i.e., the death of a wife returns her husband to the pool of unmated singles).
3. A female harem member departs from the union, with divorce rate *d*. We assume that only females leave, which seems biologically plausible. Her departure returns one female to $f_2$ and shrinks the union from $u_i$ to $u_{i-1}$. For the union $u_1$, divorce dissolves the union and returns one male to $m_2$ and one female to $f_2$.

**Fig. 6:**
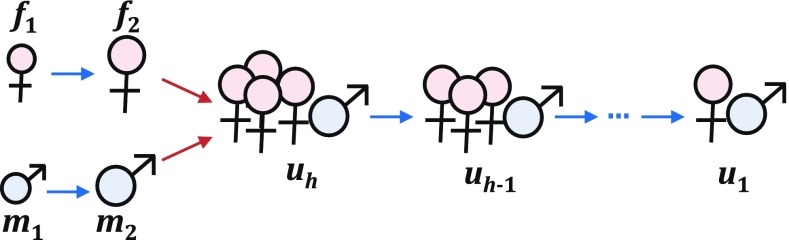
Stages in a population with maximum harem size *h*, which include juveni le males $m_1$ and juvenile females $f_1$, adult males $m_2$ and adult females $f_2$, and reproducing unions $u_i$ (one male with a harem of *i* females). Adults form harems of size *h* when mating, and these harems can shrink in size, but not grow, over time

As a result, a union may shrink (but not grow) in size due to the departure or death of its members (Fig. 6). After a union shrinks to the smallest possible size $h=1$, or if the male harem leader dies, the union dissolves and its members return to the stages for unmated adults.

Appendix 3 shows how to write the rate matrices $\mathbf {U}$, $\mathbf {B}$, and $\mathbf {T}$ for a polygynous system with maximum harem size *h*. The population vector and matrices for the case where $h=3$ are as follows:

22 $$\begin{aligned} \mathbf {n}(t)&= \begin{pmatrix} m_1&m_2&f_1&f_2&u_1&u_2&u_3 \end{pmatrix}^{{\tiny \mathsf T}}\end{aligned}$$

23 $$\begin{aligned} \mathbf {U}&= \left( \begin{matrix} 0 &{} 0 &{} 0 &{} 0 &{} 0 &{} 0 &{} 0 \\ 0 &{} -U_m &{} 0 &{} 0 &{} 0 &{} 0 &{} 0 \\ 0 &{} 0 &{} 0 &{} 0 &{} 0 &{} 0 &{} 0 \\ 0 &{} 0 &{} 0 &{} -3 U_f &{} 0 &{} 0 &{} 0 \\ 0 &{} 0 &{} 0 &{} 0 &{} 0 &{} 0 &{} 0 \\ 0 &{} 0 &{} 0 &{} 0 &{} 0 &{} 0 &{} 0 \\ 0 &{} \frac{1}{2}U_m &{} 0 &{} \frac{1}{2}U_f &{} 0 &{} 0 &{} 0 \\ \end{matrix}\right) \end{aligned}$$

24 $$\begin{aligned} \mathbf {B}&= \left( \begin{matrix} 0 &{} 0 &{} 0 &{} 0 &{} k s_1 &{} 2 k s_1 &{} 3 k s_1\\ 0 &{} 0 &{} 0 &{} 0 &{} 0 &{} 0 &{} 0 \\ 0 &{} 0 &{} 0 &{} 0 &{} k(1-s_1) &{} 2 k(1-s_1) &{} 3 k(1-s_1)\\ 0 &{} 0 &{} 0 &{} 0 &{} 0 &{} 0 &{} 0 \\ 0 &{} 0 &{} 0 &{} 0 &{} 0 &{} 0 &{} 0 \\ 0 &{} 0 &{} 0 &{} 0 &{} 0 &{} 0 &{} 0 \\ 0 &{} 0 &{} 0 &{} 0 &{} 0 &{} 0 &{} 0 \end{matrix}\right) \end{aligned}$$

25 $$\begin{aligned} \mathbf {T}&= \left( \begin{matrix} -(\mu _{m1}+\alpha _m) &{} 0 &{} 0 &{} 0 &{} 0 &{} 0 &{} 0\\ \alpha _m &{} -\mu _{m2} &{} 0 &{} 0 &{} \mu _{f2} + d &{} 0 &{} 0\\ 0 &{} 0 &{} -(\mu _{f1}+\alpha _f) &{} 0 &{} 0 &{} 0 &{} 0\\ 0 &{} 0 &{} \alpha _f &{} -\mu _{f2} &{} \mu _{m2}+ d &{} 2\mu _{m2}+d &{} 3\mu _{m2}+d \\ 0 &{} 0 &{} 0 &{} 0 &{} -(\mu _{m2} + \mu _{f2} + d) &{} \mu _{f2}+d &{} 0 \\ 0 &{} 0 &{} 0 &{} 0 &{} 0 &{} -(\mu _{m2} + \mu _{f2} + d) &{} \mu _{f2}+d \\ 0 &{} 0 &{} 0 &{} 0 &{} 0 &{} 0 &{} -(\mu _{m2} + \mu _{f2} + d)\\ \end{matrix}\right) \end{aligned}$$

**Fig. 7:**
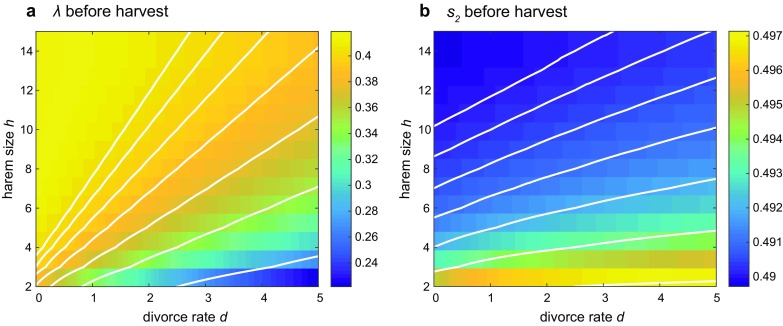
Unharvested population dynamics in the polygynous ($h>1$) union model, as functions of the divorce rate *d* and harem size *h*. *a)* Population growth rate $\lambda$. *b)* Secondary sex ratio $s_2$ (proportion of males in the adult population). Other parameters are the same as in Fig. 4

Figure 7 shows how the unharvested population rate of growth and secondary sex ratio vary across different values of *d* and *h*. As in the monogamous case, lower divorce rates result in more mated reproducing adults and, thus, higher population growth. Larger harems lead to more rapid population growth, possibly because of their higher total reproductive rates. Unions with a high maximum harem size are more resilient to divorce and female mortality, because they can lose more females before shrinking to a $u_1$ and then dissolving.

Even without sex-biased harvest, the secondary sex ratio is slightly female-biased ($s_2 \approx 0.494$), but varies only a few tenths of a percentage point across a wide range of *h* and *d*. Populations with high *h* and low *d* (large, persistent harems) are the most biased.

**Fig. 8:**
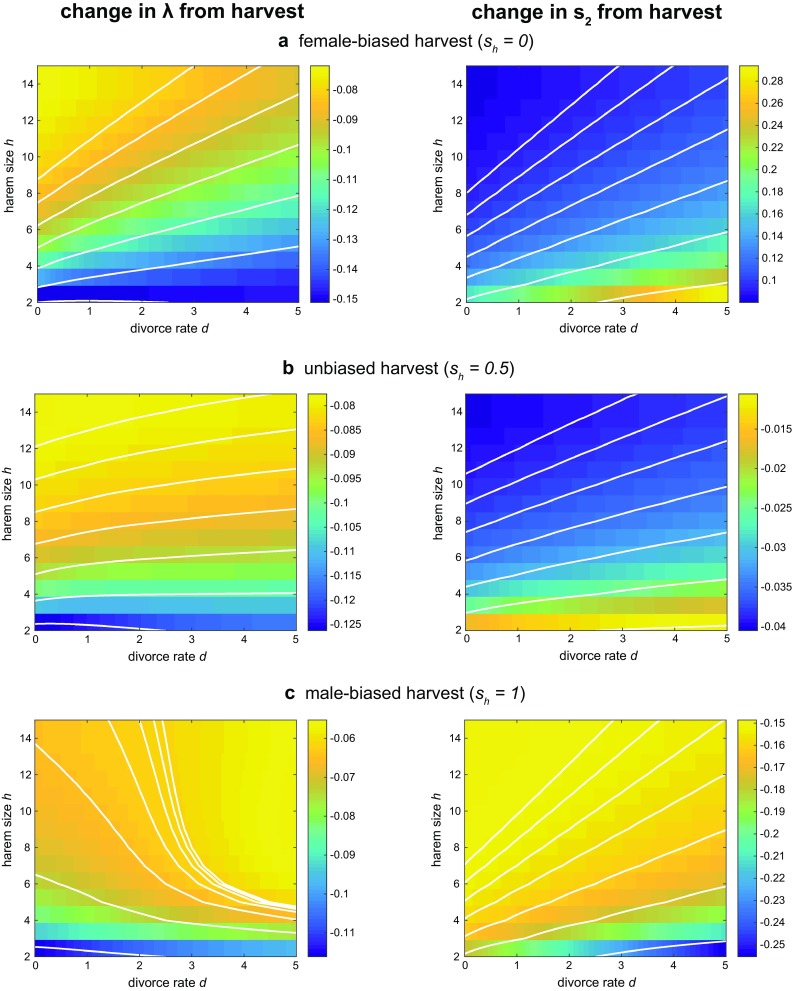
Population responses to harvest that is **a** female-biased ($s_h=0$), **b** unbiased ($s_h=0.5$), and **c** male-biased ($s_h=1$) in the polygynous ($h>1$) model. (Left) The change in population growth rate $\lambda$. (Right) The change in secondary sex ratio $s_2$. Other parameters are the same as in Fig. 4

Figure 8 demonstrate how female-biased ($s_h =0$), unbiased ($s_h =0.5$), and male-biased ($s_h =1$) harvest strategies affect population growth rates and secondary sex ratios.

1. *Female-biased harvest* (Fig. 8a) most strongly reduces growth in populations with large *d* and small *h*, the same populations with the lowest unharvested growth rates (Fig. 7a). Smaller harems are less resilient to female-biased harvest for the same reason they are less resilient to divorce and female mortality - because they cannot lose as many females before dissolving. Female-biased harvest may reduce the average harem size, and also makes it difficult for large harems to form or reform after breaking up. When *h* is high, a higher proportion of females is needed to maximize the mating rate, in accordance with Eq. 21, but these females are depleted by harvest. Increasing the divorce rate increases the rate at which harems dissolve. This more drastically reduces growth for larger harems (contours are steeper at large *h*), because they need more females to reform.
2. *Unbiased harvest* (Fig. 8b) yields similar qualitative trends. Again, populations with higher divorce rates experience greater reductions in growth, and larger harems are more affected by divorce. The effect of increasing divorce is not as pronounced as with female-biased harvest (contours are flatter overall), as less female harvest makes it easier for harems to reform. At low *h*, however, populations with lower *d* are actually more impacted by harvest. Low *h* unions have only a few females and are more likely to dissolve from increased mortality. Unions with high divorce rates are already dissolving quickly, regardless of harvest mortality. Unions with low divorce rates, in contrast, break up much more frequently once harvest mortality occurs. As a result, low *d*, low *h* populations experience the largest decreases in growth.
3. *Male-biased harvest* (Fig. 8c) reverses the effects of increased divorce rate. Focusing harvest on males is more likely to dissolve harems by killing their male leaders. Populations with low *d* experience the largest reductions in growth, because male-biased harvest makes these unions dissolve more frequently than they normally would (similar to the low *d*, low *h* case for unbiased harvest). As in the previous scenarios, the growth of large *h* populations is less affected by harvest. Even though male-biased mortality causes unions to break up more frequently, it also returns (potentially many) females to the $f_2$ pool. This may be beneficial when *h* is high, as a higher proportion of females is needed to maximize the mating rate in accordance with Eq. 21.

As expected, the secondary sex ratio $s_2$ increases during female-biased harvest, decreases during male-biased harvest, and undergoes only minimal changes when harvest is unbiased (Fig. 8, right). Populations with high *d* and low *h* experience the largest sex ratio shifts under biased harvest. This may be because the smaller growth rates of high *d*, low *h* populations are less effective in offsetting harvest-induced sex ratio biases.

**Fig. 9:**
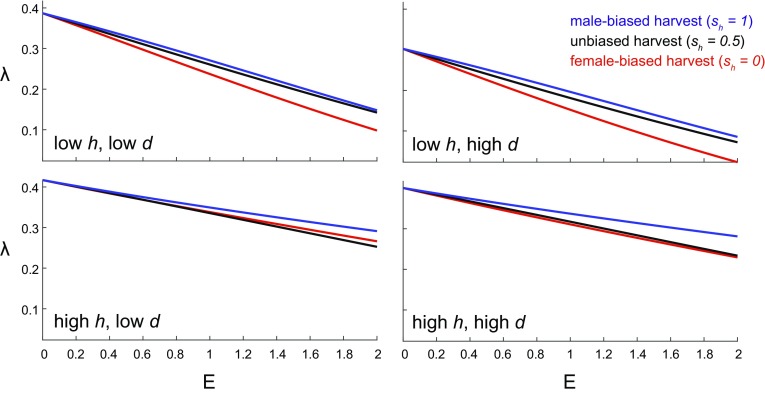
Growth rates $\lambda$ as a function of the total harvest rate *E* for populations with various mating systems. The four types of mating systems shown correspond to different harem sizes *h* and divorce rates *d* (Table 2); in this example, low $h=2$, high $h=10$, low $d=0$, and high $d=2$. Harvest may be female-biased ($s_h=0$), unbiased ($s_h=0.5$), or male-biased ($s_h=1$). Other parameters are the same as in Fig. 4

Figure 9 shows how the growth rates of the mating systems in Table 2 vary with harvest bias and intensity. Again, we see that high *h*, low *d* populations (large, persistent harems) have the largest growth rates of all the mating systems, even under harvest. Low *h*, high *d* populations (small, transient harems) have the smallest growth rates.

Increasing the total harvest rate *E* in Eq. 18 amplifies the differences between female-biased, unbiased, and male-biased harvest strategies. Female-biased harvest (red) decreases population growth more severely than male-biased harvest (blue) does, even across populations with different *h* and *d*. This may be because there is an excess of single males waiting to become harem leaders, whereas single females are usually in shorter supply (especially when *h* is large). Additionally, the death of a male harem leader immediately dissolves his union; this can return many females to the singles stage, allowing new, full-sized harems to reform with a new male leader. The death of female harem members, in contrast, does not necessarily cause the union to dissolve, and may instead generate small, stunted harems with reduced productivity.

Depending on the mating system, unbiased harvest (black) can decrease growth rates more or less than female-biased harvest does. In populations with low *h* (small harems), female-biased harvest has the most drastic impacts of all the harvest strategies — again, perhaps, because small harems cannot afford to lose as many females before dissolving. In populations with high *h* (large harems), however, unbiased harvest may be just as, if not more, detrimental to population growth.

## Discussion

The framework we present here is a tool for studying the effects of sexual reproduction, mating systems, and life histories on population dynamics. Our implementation of the BMMR approach places no limitation on the complexity of the life cycle, and is flexible enough to accommodate age or stage structure, diverse mating systems, and mating preferences. Because it is formulated as a matrix model, powerful sensitivity analysis tools are available (see Shyu and Caswell 2016a, b).

There are interesting open problems to which the approach can be applied. As formulated here, the model describes a constant environment. Time-varying (seasonal or stochastic) models would be interesting and challenging extensions. Spatial models connecting multiple subpopulations might have important effects on mate limitation. And, because the model is formulated in terms of pair formation, it will be interesting to see how it might apply to species that do not form pairs, but in which sex ratio may influence population dynamics (e.g., pollen limitation in plants).

In our application to sex-biased harvest we found that mating factors, including harem size and union duration, affect not only unharvested population growth, but also the responses of growth rate and sex ratio to sex-biased harvest. In unharvested populations, high rates of divorce, which lead to more transient unions, tend to reduce population growth, especially when harem size is small. Sex-biased harvest affects not only population sex ratios, but also long-term growth rates, with effects depending on sex bias, harem size, and divorce rate. These complex, and sometimes counterintuitive, nuances would be impossible to capture without a demographic two-sex model like this, motivating the use of such models in ecological studies.

Our two-variable depiction of the mating system, defined by harem size and union duration, can be extended to other factors. Mating systems may differ in parental investment by males and females. While polygynous males tend to provide minimal parental care, monogamous males invest on par with their female partners (Emlen and Oring 1977; Cézilly and Danchin 2008). Sex-biased harvest may have different consequences for offspring survival in these mating systems. Other species have additional nuances in how they respond to sex-biased harvest; African lions, for instance, commit infanticide when male harem leaders are killed (Whitman et al. 2004), which exacerbates the effects of male harvest on population growth.

How populations respond to selective harvest also has important consequences for evolution. Growing evidence suggests that evolutionary considerations are relevant to sustainable long-term management (Ashley et al. 2003), and that human-induced selection is especially important for harvested species. As harvest mortalities are often more severe and selective than natural mortalities, they may drive evolution in directions that would not occur under natural conditions.

Because it integrates life cycle structure, sex ratio, and a mating function, the approach introduced here is ideally suited to studying the evolution of sex ratio as a component of life history evolution. Sex ratio evolution has a long and distinguished history in evolutionary demography (e.g., Darwin 1871; Fisher 1930; Trivers 1972; Charnov 1982, among many others). Many of these discussions invoke demographic factors, such as relative mortality of males and females, but do so without a demographic model that incorporates those factors.

Our approach provides such a model, and can be analyzed using the framework of adaptive dynamics (e.g., Geritz et al. 1998), which accounts for the frequency-dependence inherent in the mating process. We have applied this to the evolution of sex ratio as influenced by sex-biased offspring costs (Shyu and Caswell 2016a) and multiple maternal conditions (Shyu and Caswell 2016b). In these studies, the dynamics of the population frequency vector $\mathbf {p}$ are used to evaluate the possibility for a mutant phenotype to invade a resident phenotype; phenotypes that are not invasible are singular strategies; their stability properties lead to the identification of evolutionarily stable (ESS) or convergence stable strategies. See Shyu and Caswell (2016a, b) for details.

### Electronic supplementary material

Below is the link to the electronic supplementary material.

Supplementary material 1 (ZIP 354 KB)

## Appendix 1: Sequential processes in continuous time

The continuous-time two sex model is obtained by imagining that the processes of mating, birth, and transitions take place as a periodic sequence, each over a discrete time interval, and then shrinking the time interval so that, in the limit, all three processes are operating at every moment. The resulting continuous-time projection matrix $\mathbf {A}$ is the average of the individual transition rate matrices in Eq. 3. Recall that the solution of a linear continuous-time system is

26 $$\begin{aligned} \frac{{\text {d}}\mathbf {n}}{{\text {d}}t} = \mathbf {A} \mathbf {n}(t) \quad \rightarrow \quad \mathbf {n}(t) = e^{t \mathbf {A}}\mathbf {n}(0) \end{aligned}$$

Thus, the matrix exponential $e^{\Delta t \mathbf {A} }$ projects the population over a discrete period of length $\Delta t$:

27 $$\begin{aligned} \mathbf {n}(t+\Delta t) = e^{\Delta t \mathbf {A} }\mathbf {n}(t) . \end{aligned}$$

Suppose that, e.g., three constant matrices $\mathbf{U}$, $\mathbf{B}$, and $\mathbf{T}$ act in sequence, over periods of length $\Delta t$. Then

28 $$\begin{aligned} \mathbf {n}(t+3\Delta t) = e^{\Delta t \mathbf {T}} e^{\Delta t \mathbf {B}} e^{\Delta t \mathbf {U}} \mathbf {n}(t) \end{aligned}$$

To first order, as $\Delta t \rightarrow 0$,

29 $$\begin{aligned} \mathbf {n}(t+3 \Delta t)&\approx (\mathbf{I}+ \Delta t \mathbf {T})(\mathbf{I}+ \Delta t \mathbf {B})(\mathbf{I}+ \Delta t \mathbf {U}) \mathbf {n}(t) \nonumber \\&\approx \left[ \mathbf{I} + \Delta t (\mathbf {T} + \mathbf {B} + \mathbf {U}) \right] \mathbf {n}(t) \end{aligned}$$

Dividing by $3\Delta t$ and taking the limit as $\Delta t \rightarrow 0$ gives the derivative,

30 $$\begin{aligned} \frac{{\text {d}}\mathbf {n}}{{\text {d}}t}&= \lim _{\Delta t \rightarrow 0} \frac{\mathbf {n}(t + 3 \Delta t) - \mathbf {n}(t)}{3 \Delta t} \nonumber \\&= \frac{1}{3} (\mathbf {T} + \mathbf {B} + \mathbf {U}) \mathbf {n}(t) \nonumber \\&= \mathbf {A} \mathbf {n}(t) \end{aligned}$$

as in Eq. 3. Heuristically, this can be thought of as a periodic product operating so fast that all three processes are operating simultaneously. In our case, the matrix $\mathbf{U}$ is a function of $\mathbf{n}(t)$, which changes over the interval $[t,t+\Delta t]$. However, as $\Delta t$ gets sufficiently small, the result is equivalent to $\mathbf{U}$ evaluated at $\mathbf{n}(t)$.

Technically, this construction is known as the product integral (Gantmacher 1959). It originated in the mathematical work of the same Vito Volterra who is recognized by ecologists for his work on population dynamics (Volterra and Hostinksy 1938).

## Appendix 2: Dynamics of the relative frequency vector $\mathbf {p}$

Given the population vector of stage frequencies

31 $$\begin{aligned} \mathbf {p} = \frac{\mathbf {n}}{ \Vert \mathbf {n} \Vert } \end{aligned}$$

the derivative $\frac{{\text {d}}\mathbf {p}}{{\text {d}}t}$ is

32 $$\begin{aligned} \frac{{\text {d}}\mathbf {p}}{{\text {d}}t}&= \frac{\Vert \mathbf {n} \Vert \frac{{\text {d}}\mathbf {n}}{{\text {d}}t} - \mathbf {n} \frac{{\text {d}}\Vert \mathbf {n} \Vert }{{\text {d}}t}}{\Vert \mathbf {n} \Vert ^2} \nonumber \\&=\frac{1}{\Vert \mathbf {n} \Vert }\frac{{\text {d}}\mathbf {n}}{{\text {d}}t} - \frac{\mathbf {n}}{\Vert \mathbf {n} \Vert ^2} \frac{{\text {d}}\Vert \mathbf {n} \Vert }{{\text {d}}t} \end{aligned}$$

Let $\mathbf {1}^{{\tiny \mathsf T}}$ be a $1 \times s$ vector of ones. Because $\mathbf {n}$ is a non-negative vector,

33 $$\begin{aligned} \Vert \mathbf {n} \Vert&= \mathbf {1}^{{\tiny \mathsf T}}\mathbf {n} \nonumber \\ \frac{{\text {d}}\Vert \mathbf {n} \Vert }{{\text {d}}t}&= \mathbf {1}^{{\tiny \mathsf T}}\frac{{\text {d}}\mathbf {n}}{{\text {d}}t} \end{aligned}$$

Then Eq. 32 can be rewritten as

34 $$\begin{aligned} \frac{{\text {d}}\mathbf {p}}{{\text {d}}t}&= \frac{1}{\mathbf {1}^{{\tiny \mathsf T}}\mathbf {n}} \frac{{\text {d}}\mathbf {n}}{{\text {d}}t} - \frac{\mathbf {n}}{(\mathbf {1}^{{\tiny \mathsf T}}\mathbf {n})^2} \mathbf {1}^{{\tiny \mathsf T}}\frac{{\text {d}}\mathbf {n}}{{\text {d}}t} \nonumber \\&= \frac{1}{\mathbf {1}^{{\tiny \mathsf T}}\mathbf {n}} \left( \mathbf {I}_s - \mathbf {p} \mathbf {I}^{{\tiny \mathsf T}}\right) \frac{{\text {d}}\mathbf {n}}{{\text {d}}t} \nonumber \\&= \frac{1}{\mathbf {1}^{{\tiny \mathsf T}}\mathbf {n}} \left( \mathbf {I}_s - \mathbf {p} \mathbf {1}^{{\tiny \mathsf T}}\right) \mathbf {A} \mathbf {n}(t) \end{aligned}$$

where $\mathbf {I}_s$ is a $s \times s$ identity matrix.

If the population is initialized with a population vector $\mathbf {n} = \mathbf {p}$ so that $\Vert \mathbf {n} \Vert = \mathbf {1}^{{\tiny \mathsf T}}\mathbf {n} = 1$, then Eq. 34 can be rewritten as:

35 $$\begin{aligned} \frac{{\text {d}}\mathbf {p}}{{\text {d}}t} = \left( \mathbf {I}_s - \mathbf {p} \mathbf {1}^{{\tiny \mathsf T}}\right) \mathbf {A} \mathbf {p}(t) \end{aligned}$$

as in Eq. 10.

## Appendix 3: BMMR matrices for a polygynous mating system

Consider a polygynous system with a maximum harem size of *h*. When *h* is large, it is cumbersome to write the rate matrices $\mathbf {U}$, $\mathbf {B}$, $\mathbf {T}$ in full, especially since many of their entries will be zeros. Instead, we will consider these matrices in terms of their contributions to these nine regions of the projection matrix:

36 $$\begin{aligned} \mathbf {A}=\left( \begin{array}{c|c|c} \mathbf {A}_{m \rightarrow m} &{} \mathbf {A}_{f \rightarrow m} &{} \mathbf {A}_{u \rightarrow m}\\ \mathbf {A}_{m \rightarrow f} &{} \mathbf {A}_{f \rightarrow f} &{} \mathbf {A}_{u \rightarrow f}\\ \mathbf {A}_{m \rightarrow u} &{} \mathbf {A}_{f \rightarrow u} &{} \mathbf {A}_{u \rightarrow u} \end{array}\right) \end{aligned}$$

For the union formation matrix $\mathbf {U}$, the only regions with nonzero contributions are:

37 $$\begin{aligned} \mathbf {U}_{m \rightarrow m}&= \begin{pmatrix} 0 &{} 0 \\ 0 &{} -U_m \end{pmatrix}&\quad 2 \times 2 \end{aligned}$$

38 $$\begin{aligned} \mathbf {U}_{f \rightarrow f}&= \begin{pmatrix} 0 &{} 0 \\ 0 &{} -h U_f \end{pmatrix}&\quad 2 \times 2 \end{aligned}$$

39 $$\begin{aligned} \mathbf {U}_{m \rightarrow u}&= \begin{pmatrix} 0 &{} 0 \\ \vdots &{} \vdots \\ 0 &{} \frac{1}{2}U_m \end{pmatrix}&\quad h \times 2 \end{aligned}$$

40 $$\begin{aligned} \mathbf {U}_{f \rightarrow u}&= \begin{pmatrix} 0 &{} 0 \\ \vdots &{} \vdots \\ 0 &{} \frac{1}{2}U_f \end{pmatrix}&\quad h \times 2 \end{aligned}$$

For the birth matrix $\mathbf {B}$, the only regions with nonzero contributions are:

41 $$\begin{aligned} \mathbf {B}_{u \rightarrow m}&= \begin{pmatrix} k s_1 &{} 2 k s_1 &{} \ldots &{} k h s_1\\ 0 &{} 0 &{} \ldots &{} 0 \end{pmatrix}&\quad 2 \times h \end{aligned}$$

42 $$\begin{aligned} \mathbf {B}_{u \rightarrow f}&= \begin{pmatrix} k (1-s_1) &{} 2 k (1-s_1) &{} \ldots &{} k h (1-s_1)\\ 0 &{} 0 &{} \ldots &{} 0 \end{pmatrix}&\quad 2 \times h \end{aligned}$$

For the transition matrix $\mathbf {T}$, the only regions with nonzero contributions are:

43 $$\begin{aligned} \mathbf {T}_{m \rightarrow m}&= \begin{pmatrix} -(\mu _{m1} + \alpha _m) &{} 0 \\ \alpha _m &{} -\mu _{m2} \end{pmatrix}&\quad 2 \times 2 \end{aligned}$$

44 $$\begin{aligned} \mathbf {T}_{f \rightarrow f}&= \begin{pmatrix} -(\mu _{f1} + \alpha _f) &{} 0 \\ \alpha _f &{} -\mu _{f2} \end{pmatrix}&\quad 2 \times 2 \end{aligned}$$

45 $$\begin{aligned} \mathbf {T}_{u \rightarrow m}&= \begin{pmatrix} 0 &{} 0 &{} \ldots &{} 0\\ \mu _{f2}+d &{} 0 &{} \ldots &{} 0 \end{pmatrix}&\quad 2 \times h \end{aligned}$$

46 $$\begin{aligned} \mathbf {T}_{u \rightarrow f}&= \begin{pmatrix} 0 &{} 0 &{} \ldots &{} 0\\ \mu _{m2}+d &{} 2\mu _{m2}+d &{} \ldots &{} h \mu _{m2}+d \end{pmatrix}&\quad 2 \times h \end{aligned}$$

The $h \times h$ submatrix $\mathbf {T}_{u \rightarrow u}$ is also nonzero. It contains entries of $-(\mu _{m2} + \mu _{f2} + d)$ all along its diagonal, and entries of $\mu _{f2} + d$ all along its first superdiagonal.

As an example, the $\mathbf {U}$, $\mathbf {B}$, $\mathbf {T}$ matrices in the case where $h=3$ are provided in Eqs. 23, 24, and 25 respectively.

## Appendix 4: Multiple mating stages and mating preferences

Suppose^1^ that there are *q* adult male stages and *r* adult female stages. There are *qr* types of unions possible. The rate of production of (*i*, *j*) matings depends on males of stage *i* and females of stage *j*, and on preferences. In this case, it is a particular advantage to work with a continuous time model because it specifies the rates of mating rather than probabilities; to specify probabilities would require accounting for probabilities of events that did not happen.

The mating process, where adult males and females pair into reproducing unions, is described by the union formation matrix $\mathbf {U}$. Mating functions in $\mathbf {U}$ give the rates of union formation as functions of the relative frequencies of males and females available to mate, and are thus functions of the stage frequency vector $\hat{\mathbf {p}}$.

Mating preferences in the mating functions describe the probabilities of favoring partners of certain conditions. The female preference distribution $g_j(i)$ gives the proportion of Condition *j* females that mate with Condition *i* males. Similarly, the male preference distribution $h_i(j)$ gives the proportion of Condition *i* males that mate with Condition *j* females. Summing these distributions over all male and female conditions respectively yields a total probability of 1:

47 $$\begin{aligned} \sum _i g_j(i)&= 1 \quad \forall \; j\end{aligned}$$

48 $$\begin{aligned} \sum _j h_i(j)&= 1 \quad \forall \; i \end{aligned}$$

Examples of mating preference distributions include:

1. Fully assortative mating, where individuals only mate with partners in the same condition:
  49 $$\begin{aligned} g_j(i)&= 1 \; \text {if i = j, 0 else} \nonumber \\ h_i(j)&= 1 \; \text {if i = j, 0 else} \end{aligned}$$
2. Random mating, where individuals pick partners based on their relative abundances in the population:
  50 $$\begin{aligned} g_j(i)&= \frac{m_i}{\sum _i m_i} \nonumber \\ h_i(j)&= \frac{f_j}{\sum _j f_j} \end{aligned}$$
3. Biased mating, where individuals prefer partners of certain conditions. An attractiveness or competitiveness factor $c_i$ weighs the abundance of each partner condition, e.g.,:
  51 $$\begin{aligned} g_j(i)&= \frac{c_i m_i}{\sum _i c_i m_i} \nonumber \\ h_i(j)&= \frac{c_j f_j}{\sum _j c_j f_j} . \end{aligned}$$
  Partners with larger $c_i$ are more preferable mates. If all $c_i$ are equal, Eq. 51 reduces to the random mating case Eq. 50. If $c_i=0$, individuals of stage *i* do not mate.

The total mating function $M_{ij}(\mathbf {n})$ gives total unions $u_{ij}$ (Condition *i* males mated with Condition *j* females) formed per time. The most general and flexible mating functions are based on generalized weighted means (Hölder means). These have the general form:

52 $$\begin{aligned} M_{ij}(\mathbf {n}) = \left( b [f_j g_j(i)]^a + (1-b) [m_i h_i(j)]^a \right) ^{1/\alpha} \end{aligned}$$

where $0\le b \le 1$ and $a < 0$ (Hadeler 1989; Martcheva and Milner 2001; Caswell 2001). Note that $M_{ij}(\mathbf {n})$ is calculated only over individuals that are available to mate (i.e., adult single male stages $m_i$ and adult single female stages $f_j$). As a result, the mating function does not depend on the males and females in non-mating stages, such as immature juveniles or adults already in unions.

The harmonic mean mating function is one of the most widely used, because it satisfies the biological criteria for two sex models and captures the qualitative properties of a wide range of Holder means (Caswell and Weeks 1986; Iannelli et al. 2005). Hence, we use a harmonic mean mating function where $a= -1, b = 1/2$, so that:

53 $$\begin{aligned} M_{ij}(\mathbf {n}) = \frac{2 m_i h_i(j) f_j g_j(i)}{ m_i h_i(j) + f_j g_j(i)} . \end{aligned}$$

The corresponding male and female per capita mating functions are:

54 $$\begin{aligned} U_{m,ij}(\mathbf {n})&= \frac{M_{ij}(\mathbf {n})}{m_i} \nonumber \\ U_{f,ij}(\mathbf {n})&= \frac{M_{ij}(\mathbf {n})}{f_j} . \end{aligned}$$
