# Supplementary material for: Mating, births, and transitions: a flexible two-sex matrix model for evolutionary demography
Source: Popul Ecol. 2018 Jun 1;60(1):21–36. doi: 10.1007/s10144-018-0615-8 (PMC6435235; doi:10.1007/s10144-018-0615-8)
Supplement: Supplementary file 1 — Supplementary material 1 (ZIP 354 KB) [file 10144_2018_615_MOESM1_ESM.zip › Shyu and Caswell Supplement MATLAB code/Readme.pdf]

E. Shyu and H. Caswell. Matings, births, and transitions: a flexible two-sex matrix model for evolutionary demography.

Matlab code for examples

This .zip file contains MATLAB code for the example calculations in the paper. Each MATLAB script corresponds to one of the figures in the paper.

* Figure 3 (Dynamics of the 5-stage BMMR model): dynamics.m

* Figure 4 (Population dynamics and responses to harvest in the monogamous model): harvestplots_monogamy.m

* Figures 7 and 8 (Population dynamics and responses to harvest in the polygynous model): harvestplots.m

* Figure 9 (Responses to harvest for other mating systems): matesys_plots.m

These scripts make use of the following functions, which are also provided as files.

count_adults.m
Calculates the secondary sex ratio s_2, as the proportion of all adults that are male.

count_mated_adults.m
Calculates the proportion of all adults that are mated (in unions).

find_clevels.m
Calculates contours that are evenly spaced.

harem_invfit_p.m
Integrates dp/dt until the frequency vector p converges. Similar to harem_invfit.m, which is for the population vector n.

harem_invit.m
Integrates dn/dt until the population vector n converges.

harem_ODE_p.m
dp/dt, integrated by harem_invfit_p.m

harem_symbolicsetup.m
Sets up the matrices and derivatives symbolically. Initial structure is similar to harem.m.
