# Supplementary material for: Mating, births, and transitions: a flexible two-sex matrix model for evolutionary demography
Source: Popul Ecol. 2018 Jun 1;60(1):21–36. doi: 10.1007/s10144-018-0615-8 (PMC6435235; doi:10.1007/s10144-018-0615-8)
Supplement: Supplementary file 1 — Supplementary material 1 (ZIP 354 KB) [file 10144_2018_615_MOESM1_ESM.zip › Shyu and Caswell Supplement MATLAB code/Readme.doc]

E. Shyu and H. Caswell. Matings, births, and transitions: a flexible two-sex matrix model for evolutionary demography.

**Matlab code for examples**

This .zip file contains MATLAB code for the example calculations in the paper. Each MATLAB script corresponds to one of the figures in the paper.

* Figure 3 (Dynamics of the 5-stage BMMR model): dynamics.m

* Figure 4 (Population dynamics and responses to harvest in the monogamous model): harvestplots_monogamy.m

* Figures 7 and 8 (Population dynamics and responses to harvest in the polygynous model): harvestplots.m

* Figure 9 (Responses to harvest for other mating systems): matesys_plots.m

These scripts make use of the following functions, which are also provided as files.

count_adults.m

Calculates the secondary sex ratio s_2, as the proportion of all adults that are male.

count_mated_adults.m
Calculates the proportion of all adults that are mated (in unions).

find_clevels.m
Calculates contours that are evenly spaced.

harem_invfit_p.m

Integrates dp/dt until the frequency vector p converges. Similar to harem_invfit.m, which is for the population vector n.

harem_invit.m
Integrates dn/dt until the population vector n converges.

harem_ODE_p.m
dp/dt, integrated by harem_invfit_p.m

harem_symbolicsetup.m

Sets up the matrices and derivatives symbolically. Initial structure is similar to harem.m.
